# Anti-Inflammatory, Antioxidant, and Anti-Atherosclerotic Effects of Natural Supplements on Patients with FMF-Related AA Amyloidosis: A Non-Randomized 24-Week Open-Label Interventional Study

**DOI:** 10.3390/life12060896

**Published:** 2022-06-15

**Authors:** Micol Romano, Facundo Garcia-Bournissen, David Piskin, Ulkumen Rodoplu, Lizzy Piskin, Abdelbaset A. Elzagallaai, Tunc Tuncer, Siren Sezer, Didar Ucuncuoglu, Tevfik Honca, Dimitri Poddighe, Izzet Yavuz, Peter Stenvinkel, Mahmut Ilker Yilmaz, Erkan Demirkaya

**Affiliations:** 1 Department of Paediatrics, Division of Paediatric Rheumatology, Schulich School of Medicine & Dentistry, University of Western Ontario, London, ON N6A 5W9, Canada; micol.romano@lhsc.on.ca (M.R.); erkan.demirkaya@lhsc.on.ca (E.D.); 2 Canadian Behcet and Autoinflammatory Disease Center (CAN BE AID), Schulich School of Medicine & Dentistry, University of Western Ontario, London, ON N6A 5W9, Canada; david.piskin@lhsc.on.ca; 3 Department of Paediatrics, Division of Paediatric Clinical Pharmacology, Schulich School of Medicine & Dentistry, University of Western Ontario, London, ON N6A 5W9, Canada; facundo.garcia-bournissen@lhsc.on.ca; 4 Department of Epidemiology and Biostatistics, Schulich School of Medicine & Dentistry, University of Western Ontario, London, ON N6A 3K7, Canada; 5 Emergency Medicine Association of Turkey of All, 35220 Izmir, Turkey; ulkumenrodoplu@yahoo.com; 6 Robarts Research Institute, University of Western Ontario, London, ON N6A 3K7, Canada; rerpiski@uwo.ca; 7 Schulich School of Medicine & Dentistry, Physiology and Pharmacology, University of Western Ontario, London, ON N6A 3K7, Canada; aelzaga@uwo.ca; 8 Unit of Biochemistry, Epigenetic Health Solutions, 06810 Ankara, Turkey; tuncmember@yahoo.com; 9 Division of Nephrology, Faculty of Medicine, Atilim University, 06830 Ankara, Turkey; siren.sezer@atilim.edu.tr; 10 Department of Food Engineering, Faculty of Engineering, Cankiri Karatekin University, 18100 Cankiri, Turkey; didaru@karatekin.edu.tr; 11 Unit of Biochemistry, Gur Life Hospital, 26320 Eskisehir, Turkey; drth16@gmail.com; 12 Department of Medicine, Nazarbayev University School of Medicine, Nur-Sultan 010000, Kazakhstan; dimitri.poddighe@nu.edu.kz; 13 Clinical Academic Department of Pediatrics, National Research Center of Maternal and Child Health, University Medical Center, Nur-Sultan 010000, Kazakhstan; 14 Department of Nephrology, Lokman Hekim University, 06510 Ankara, Turkey; nefrolife35@yahoo.com; 15 Department of Renal Medicine M99, Karolinska Institute, Karolinska University Hospital, 17164 Stockholm, Sweden; peter.stenvinkel@ki.se; 16 Unit of Nephrology, Center for Epigenetic Health Solutions, 06810 Ankara, Turkey

**Keywords:** familial Mediterranean fever, AA amyloidosis, natural supplementation, endothelial dysfunction, oxidative stress, inflammation

## Abstract

We aimed to evaluate the effect of a combination of natural products on parameters related to inflammation, endothelial dysfunction, and oxidative stress in a cohort of familial Mediterranean fever (FMF) patients with Serum Amyloid A amyloidosis, in a non-randomized, 24-week open-label interventional study. *Morinda citrifolia* (anti-atherosclerotic-AAL), omega-3 (anti-inflammatory-AIC), and extract with Alaskan blueberry (antioxidant-AOL) were given to patients with FMF-related biopsy-proven AA amyloidosis. Patients were >18 years and had proteinuria (>3500 mg/day) but a normal estimated glomerular filtration rate (eGFR). Arterial flow-mediated dilatation (FMD), carotid intima media thickness (CIMT), and serum biomarkers asymmetric dimethylarginine (ADMA), high sensitivity C-reactive protein (hs-CRP), pentraxin (PTX3), malondialdehyde (MDA), Cu/Zn-superoxide dismutase (Cu/Zn-SOD), and glutathione peroxidase (GSH-Px) were studied at baseline and after 24 weeks of treatment. A total of 67 FMF-related amyloidosis patients (52 male (77.6%); median age 36 years (range 21–66)) were enrolled. At the end of a 24-week treatment period with AAL, AIC, and AOL combination therapy, ADMA, MDA, PTX3, hsCRP, cholesterol, and proteinuria were significantly decreased compared to baseline, while CuZn-SOD, GSH-Px, and FMD levels were significantly increased. Changes in inflammatory markers PTX3, and hsCRP were negatively correlated with FMD change, and positively correlated with decreases in proteinuria, ADMA, MDA, cholesterol, and CIMT. Treatment with AAL, AIC and AOL combination for 24 weeks were significantly associated with reduction in inflammatory markers, improved endothelial functions, and oxidative state. Efficient control of these three mechanisms can have long term cardiovascular and renal benefits for patients with AA amyloidosis.

## 1. Introduction

Familial Mediterranean fever (FMF) is an autosomal recessive autoinflammatory disorder seen mainly in descendants from Mediterranean populations such as Jews, Arabs, Turks, and Armenians [1]. Common clinical symptoms are abdominal pain, joint involvement (arthralgia or arthritis), and chest pain accompanied by fever. Elevated acute phase reactants are seen during the active episodes but return to reference values in attack-free periods. However, partial responders or non-responders may have ongoing sub-clinical inflammation during the asymptomatic period, and those patients may develop secondary amyloidosis due to increased serum amyloid A (SAA) protein [2]. AA amyloidosis causes chronic kidney disease, and it is the most serious morbidity in untreated or non-responder FMF patients.

Evidence indicates increased oxidative stress in patients with FMF [3,4,5,6,7]. Oxidative stress is shown as one of the reasons for endothelial dysfunction (ED) and atherosclerosis [8]. Lipid peroxidation (LP) causes the formation of reactive hydroperoxides and their subsequent decomposition products, such as malondialdehyde (MDA). MDA has been shown to be an accurate biomarker for oxidative stress in patients with FMF [3,4,8,9]. Increased carotid artery intima media thickness (CIMT) [10,11] and impaired flow-mediated dilatation (FMD) have been reported in FMF patients [10,11,12]. High levels of asymmetric dimethyl arginine (ADMA) reflect ED in FMF [10,11,12]. A positive correlation between SAA, fibrinogen, erythrocyte sedimentation rate, and CIMT has been observed, supporting the idea that inflammation begets atherosclerotic lesion development [13].

FMF is characterized by spontaneous episodes of activation of the inflammasome, resulting in IL-1 beta release, which has been associated with deleterious cardiometabolic outcomes [14,15]. Patients with AA amyloidosis are secondary to FMF and progress to chronic renal failure. Chronic inflammation due to FMF and renal failure cause a high mortality rate in FMF-related AA amyloidosis because of the cardiac complications [11,16,17]. In a previous report, we showed that cardiovascular disease (CVD) related to mortality rates are higher in patients with AA amyloidosis, secondary to FMF which was assignable to inflammation and vascular dysfunction [18]. It can be hypothesized that better management provides efficient control of inflammation, diminishing the CVD risk.

Since inflammation, oxidative stress, and endothelial dysfunction jointly contribute to morbidity and mortality in this patient group, our approach is to modulate these pathways by using a combination of health supplements with anti-atherosclerotic properties (containing *Morinda citrifolia*) [19,20], anti-inflammatory properties (omega-3) [21], and anti-oxidant properties (extract of Alaskan blueberry and 21 different red and purple fruit vegetables) [22,23]. These preparations were previously shown to decrease inflammatory markers and improve endogenous defenses against oxidative injury and lipid peroxidation when used for 12 weeks in our previous study [24]. Our previous work also supported treatment with this combination to improve endothelial function in patients with different chronic diseases [24].

The objective of the current study is to evaluate changes in surrogate markers of inflammation, oxidative stress, and endothelial function following 24 weeks of AAL, AIC, and AOL therapies. We analyze the effect of AAL, AIC, and AOL supplementation on biochemical and radiologic parameters in patients with FMF-related AA amyloidosis.

## 2. Materials and Methods

### 2.1. Patients

**Figure 1:**
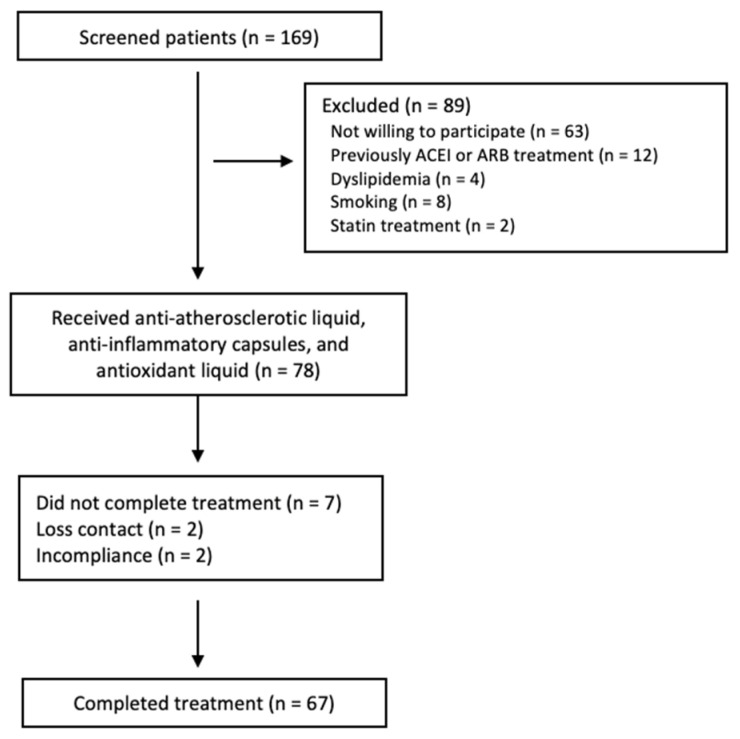
Flow chart of patients enrolled in the trial.

Patients with a diagnosis of AA amyloidosis secondary to FMF were enrolled and followed at a center. There were 169 patients who were followed with the diagnosis of FMF-related amyloidosis (Figure 1) [25]. Tel–Hashomer criteria were used for the diagnosis of FMF [26]. A kidney biopsy diagnosed FMF-related amyloidosis with Congo red dye. We included patients with FMF-related amyloidosis and met the following criteria: >18 years, with systolic blood pressure ≤ 140 mmHg and/or diastolic blood pressure ≤ 90 mmHg. All patients recruited to the study had a glomerular filtration rate within the normal range (eGFR) (≥90 mL/min/1.73 m^2^). Patients currently or previously treated with an angiotensin-converting enzyme inhibitors (ACEI) or angiotensin receptor blockers (ARBs) or statins were excluded. Patients were also excluded if they had other risk factors such an obesity (BMI > 30 kg/m^2^), dyslipidemia (total cholesterol > 280 mg/dL, fasting triglycerides >180 mg/dL), renal failure (eGFR < 90 mL/min/1.73 m^2^) or smoking.

The protocol was approved by the local Ministry of Health. All study procedures were in compliance with the WMA Declaration of Helsinki principles, as well as local laws. All patients included in the study provided informed consent prior to participation in the study. We followed the “Improving the reporting quality of nonrandomized evaluations of behavioural and public health interventions: TREND” statement checklist [27]. All patients were enrolled during the period 1 December 2018–1 March 2021.

### 2.2. Intervention

We conducted a prospective, interventional, open-label study to evaluate the effect of a combination of *Morinda citrifolia* anti-atherosclerotic liquid (AAL), omega-3 anti-inflammatory capsules (AIC), and Alaskan blueberry and 21 different red and purple fruit vegetables anti-oxidant liquid (AOL) on inflammatory markers, endothelial function markers, and redox status in patients with FMF-related amyloidosis [28]. Patients were treated with AAL, 3 mL once per day (Nitro, Kyani, Idaho Falls, ID, USA)), AIC, 3 capsules, 2535 mg once per day (Sunset, Kyani, Idaho Falls, ID, USA), and an AOL, 30 mL once per day (Sunrise, Kyani, Idaho Falls, ID, USA) for a total of 24 weeks immediately following baseline measurements. The natural compounds used were obtained from a commercial source (Kyani, Idaho Falls, ID, USA) to ensure consistency in doses among patients. The dosage used was the amount recommended by the manufacturer. 

Blood samples were drawn from patients every two weeks during the study period. Serum creatinine and potassium were measured. The dose of AAL, AIC, and AOL were adjusted to achieve serum potassium levels of <5.5 mEq/L. All patients continued to receive all treatments for their disease that they were taking at baseline. All the patients who were recruited for the protocol were instructed not to use any other vitamins or supplements.

### 2.3. Study Endpoints

The FMD percentage change at the 24th week of the study was the primary endpoint compared to baseline. Secondary endpoints included changes in proteinuria, carotid intima media thickness (CIMT), inflammatory markers (hsCRP, SAA, PX3), endothelial biomarkers, and the serum lipid profile.

### 2.4. Clinical and Cardiovascular Assessment

Study patients underwent detailed clinical examination, chest X-ray, baseline electrocardiography, ECHO (2D), transaminases levels, renal function tests, and urinary protein excretion (24-h). Morning blood pressure while resting was measured on three occasions from the right arm using mercury sphygmomanometer and recorded as mean values for diastolic and systolic blood pressures.

### 2.5. Assessment of Endothelial Dysfunction

We used the same protocol which was described in detail in our preliminary study [24]. Briefly, endothelial dysfunction was investigated as per the method of Celemajer et al. [29]. A single observer obtained all the measurements using an ATL 5000 ultrasound system with a 12-Mhz probe (Advanced Technology Laboratories Inc., Bothell, WA, USA). All vasoactive treatments were discontinued 24 h before assessment. Ultrasound images were recorded for further blind analysis, which was subsequently performed. After reactive hyperemia, three measurements were performed consecutively to measure the maximum diameter and average value calculated to define FMD diameter. The percent change was calculated between the measured diameters of baseline at the end of the study period for the comparisons of FMD.

### 2.6. Carotid-Intima-Media Thickness (CIMT)

Ultrasonographic measurements on a common carotid artery were performed on both sides using a high-resolution Doppler ultrasound (ATL 5000) with a 5–12 MHz linear transducer. All measurements were performed by a single-blinded technician on two recorded longitudinal images of each artery. The average value of the four measurements was calculated to define CIMT.

### 2.7. Laboratory Measurements

**Blood chemistry:** All samples were obtained from subjects after twelve hours of fasting. Prior to the blood sample collection, patients were instructed to abstain from physical activity for at least half an hour. Laboratory investigations including cholesterol (TC), triglyceride (TG), high-density lipoprotein cholesterol (HDL), fasting plasma glucose (FPG), serum ADMA, MDA, CuZn-SOD, GSH-Px, hsCRP, PTX3 levels, and basal insulin values were performed for all patients at baseline [24], and after the intervention period [30]. SAA was measured in serum with latex nephelometry (BNII autoanalyzer; N Latex SAA Siemens Healthcare Diagnostics, Germany). The lower detection limit was 0.8 mg/L, with an intra-assay coefficient of variation (CV) of 4.7% and intra-assay coefficient of variation (CV) of 6.2%.

**Erythrocyte antioxidant capacity:** Blood samples were obtained in heparinized polypropylene tubes after twelve hours of fasting. Plasma and erythrocytes were separated from blood samples and used to measure trace elements and enzyme activities for oxidative stress. Erythrocyte CuZn-SOD and GSH-Px activity was measured in a UV–Vis Recording Spectrophotometer (UV-2100S; Shimadzu Co., Kyoto, Japan) as described in our previous study [24,31]. 

**Erythrocyte MDA level measurement:** Erythrocyte MDA levels were measured on an erythrocyte lysate obtained after centrifugation and in accordance with the method previously described by Jain [32]. MDA levels were measured spectrophotometrically using a thiobarbituric acid reagent. A known concentration of tetrametoxypropane solution was used as a standard. The levels of MDA were expressed as nmol/mL.

### 2.8. Statistical Methods

Normality distribution of the variables was assessed with graphical methods and Kolmogorow–Smirnov test. Irregular distributed variables were expressed as median (range) and normally distributed variables as mean ± SD. Paired sample test and Wilcoxon test were used where appropriate to compare before and after measurements. Pearson’s correlation analysis was used to determine correlations between the changes in FMD, CIMT, MDA, ADMA, hsCRP, PTX3, cholesterol, and proteinuria. A *p*-value < 0.05 was considered to be statistically significant. All statistical analyses were performed by using SPSS 21.0 (SPSS Inc., Chicago, IL, USA) statistical package. 

## 3. Results

### 3.1. Baseline Characteristics

A total of 67 patients (52 male (77.6%)) were used to complete the study (Figure 1). All patients had nephrotic-range proteinuria (24 h protein excretion >3500 mg/day) and normal eGFR. The median age at enrollment was 36 years (range 21–66 years). The most frequent mutated alleles were M694V, M680I, and E148Q (Table 1). M694V mutation was found in almost half of patients (n = 34, 50.7%) in homozygosity. There were 55.2% (n = 37) patients with homozygous mutations in the MEFV gene, 40.2% (n = 27) of patients were compound heterozygotes, and 4.4% (n = 3, all M694V/-) patients with heterozygote genotype status. In this cohort, 13.4% of the studied population presented with a family history of FMF. At the baseline assessment, the most frequent symptoms were fever (88.1%), abdominal pain (68.7%), arthritis (67.2%), arthralgia (38.8%), and chest pain 33 (50.3%) in patients with AA amyloidosis secondary to FMF (Table 2). All patients reported that they had been treated with colchicine, and the majority of them (83.6%) were taking colchicine at the time of the recruitment period. 

### 3.2. Effect of AAL, AIC, and AOL on FMD and Inflammation

After 24 weeks of combined treatment of AAL, AIC, and AOL, a 25% increase in FMD (i.e., an absolute 1.3%) (*p* < 0.001) was observed (Table 3). The endothelial function surrogate biomarker ADMA also showed a statistically significant improvement, and clinical and subclinical biomarkers such as hsCRP, PTX3, and SAA significantly decreased after treatment with AAL, AIC, and AOL compared to baseline (Table 3). CIMT, MDA, and cholesterol levels decreased significantly compared to baseline, and biomarkers of endogenous defenses against oxidative injury, CuZn-SOD, and GSH-Px, significantly elevated during the study period. Table 3 depicts changes in specific parameters in the 67 enrolled subjects. 

We observed a moderate negative correlation between change (i.e., between baseline and 24 weeks) in FMD and proteinuria (r = −0.53, *p* < 0.001), hsCRP (r = −0.47, *p* < 0.001), PTX3 (r = −0.50, *p* < 0.001), and ADMA (r = −0.49, *p* < 0.001), respectively (Table 4). Moreover, there was a moderate correlation between change in proteinuria and cholesterol (r = 0.48, *p* < 0.001), hsCRP (r = 0.41, *p* < 0.001), and ADMA (r = 0.41, *p* < 0.001) levels (Table 4). Moderate correlations between change in CIMT and MDA (r = 0.49, *p* < 0.001), hsCRP (r = 0.46, *p* < 0.001), PTX3 (r = 0.48, *p* < 0.001), and ADMA (r = 0.44, *p* < 0.001) were observed (Table 4).

## 4. Discussion

Familial Mediterranean fever (FMF) is a disease that is characterized by spontaneous episodes of activation of specific inflammatory pathways, including inflammasomes, causing IL-1B release and chronic inflammation, which drive AA amyloidosis and CKD [33]. We report the findings of an open-label 24-week trial examining the effects of a combination of bioactive nutrients on specific inflammatory, oxidative, and vascular biomarkers in a cohort of CKD stage 1 patients due to AA amyloidosis secondary to FMF. We report significant improvements in endothelial dysfunction (FMD and ADMA). In addition, a significant reduction in inflammatory markers (i.e., hsCRP and PTX3), oxidative stress, and anti-atherosclerotic markers (e.g., CIMT, MDA) were observed. Our results agree with the concept “Food as Medicine” and using bioactive nutrients to target the risk factor profile in kidney disease [34].

Homozygous M694V mutation in the *MEFV* gene causes a more severe FMF phenotype and is one of the major risk factors for AA amyloidosis [35]. In our cohort, M694V was the most frequent mutated allele, found in almost half of the patients as homozygous. Further, patients with homozygote M694V and homozygote M680I mutations could be at risk for early progression of coronary vascular events [36]. A high concentration of ADMA is associated with risk factors for atherosclerosis, ED, and CKD. It has been reported that patients with M694V/M694V mutation have significantly elevated serum ADMA levels compared to those of other genotypes [37]. However, the results of other studies were controversial to show differences between mutations [38]. Based on these observations, interventions that suppress subclinical inflammation and improve endothelial dysfunction should be considered in FMF. Three patients in our cohort were heterozygous for M694V and developed AA amyloidosis. It is important to keep in mind the possibility that patients in simple heterozygosity may present symptoms of FMF and, in that case, should be treated [39].

Endothelial dysfunction is the key mechanism driving cardiovascular disease in FMF patients complicated with AA amyloidosis [11,12]. A few studies have reported differing results related to FMD measurements to determine ED as a surrogate biomarker for CVD risk in FMF patients [11,12,40]. Patients with FMF-related amyloidosis have lower levels of FMD and higher ADMA concentrations compared to patients with primary glomerulonephritis or healthy controls [11]. Meanwhile, CIMT values have been reported to be similar [12]. Our results indicate that 24 weeks of supplementation with the combination of bioactive nutrients significantly improved FMD. Elevated plasma levels of ADMA are found in several disease states involving vascular malfunction, and ADMA elevated levels correlate with CKD progression [41,42]. The benefits of ADMA lowering therapies have been studied and reported in experimental models of vascular diseases [43]. In this study, high ADMA levels decreased after intervention with bioactive nutrients, and we report a moderate correlation between the level of changes in ADMA and FMD. Our study supports our previous findings that report a correlation between ADMA and inflammation in FMF [24]. Multiple clinical reports have revealed and suggested that ADMA is a surrogate biomarker and a contributor to vascular dysfunction and CVD. Our group recently confirmed that patients with FMF-related AA amyloidosis have an increased risk for CVD and premature mortality [18] and that hsCRP, as a marker of inflammation, and FMD, as an indicator of vascular function, are independent predictors of mortality in this group [18]. 

We report that this combination of bioactive nutrients has beneficial effects on vascular function through ameliorating inflammation (hsCRP, PTX3, and SAA). The reduction in hsCRP was associated with decreases in proteinuria and other vascular function markers. This is in agreement with experimental models that suggest that mitigations in the inflammatory response are associated with decreased kidney injury and improved renal functions [44].

The biological basis for the increased risk of CVD in FMF has not been well established but likely relates to persistent inflammation. It has been shown that patients with AA amyloidosis due to FMF have an increased incidence of premature CVD events and death compared to other patient groups with CKD [18]. CVD preventive strategies are more cost-effective than treating the complications at a later stage and necessary to identify the risk ratio in patients with severe FMF, amyloidosis, and subclinical atherosclerosis [45]. There have been controversies on the extent of subclinical atherosclerosis (as evaluated by CIMT) in patients with FMF without amyloidosis [40]. Patients with a severe disease course or being non-adherent to colchicine may progress and develop AA amyloidosis, with resulting proteinuria and CKD [46]. In addition to the disease itself, AA amyloidosis and proteinuria additively contribute to diminished endothelial function and inflammation in the absence of CKD [47]. In the present study, we observed that the CIMT had decreased at the end of the 24-week study period compared to baseline. In this study, we screened for CIMT with the classical method used in our previous studies [48,49,50] instead of cross-sectional carotid intima media (CIM) area [51,52]. Given the short intervention time period and pronounced decrease in CIMT, these findings could be related to methodology. It is likely that the CIM area would be a better predictor and more reliable indicator.

Oxidative stress is thought to play a critical and decisive role in the pathophysiology of several disorders. The FMF episodes are characterized by activated polymorphonuclear leukocytes (PMNL) tissue infiltration, particularly in the peritoneum, joint, and pleura. The presence of increased oxidative stress during the acute FMF episodes has been documented and confirmed by an impaired antioxidant response in plasma accompanied by boosted lipid peroxidation (LP) [4,7]. Plasma antioxidant defense mechanisms that scavenge ROS include CuZn superoxide dismutase (SOD)-, catalase (CAT)-, and glutathione peroxidase (GSH-Px) [53]. At the end of the study period, MDA and cholesterol concentrations were significantly reduced compared to baseline, although CuZn-SOD, GSH-Px levels, and FMD measurements were increased. Elevated levels of MDA at the baseline might be the result of enhanced peroxidation due to a higher level of cholesterol in the existence of oxidative stress. MDA is one of the main biomarkers of lipid peroxidation, which is associated with a high risk of atherosclerosis. We found significant correlations between the MDA levels and inflammatory markers. Those changes between the baseline and 24 weeks included decreased MDA, hsCRP, and PTX3 levels, which may affect vascular functions in the long term. In this perspective, the interplay between oxidative parameters and inflammation is a crucial issue because both MDA and hsCRP/PTX3 have contributed to endothelial dysfunction and atherosclerosis progression. Our results support the concept that increased oxidative stress and inflammation beget atherosclerotic lesion development. The association between free radical activity and different kinds of amyloidosis has been shown in in vitro or in vivo reports [6,54,55] and also addressed in the pathogenesis of progression for kidney injury [56]. Gurbuz et al. reported increased plasma lipid peroxidation and MDA levels in FMF patients with proteinuria [4]. The same group also found significantly higher MDA concentrations in FMF patients with proteinuria compared to the patients without proteinuria. Interestingly, there were no differences between the two groups with respect to levels of antioxidant enzymes. Our results support that the beneficial effects of this combination of bioactive nutrients are mainly related to their free radical savaging and anti-inflammatory activities.

Although subclinical atherosclerosis is more frequent in FMF patients, atherosclerotic cardiovascular disease was less prevalent than the healthy controls in Israel [57]. This unexpected finding has been interpreted as a consequence of colchicine usage, which reduces cardiovascular risks in pre-clinical and clinical studies, possibly by decreasing neutrophil activation [57]. Neutrophils are primary cell types playing a major role in the pathophysiology of FMF [7], and responsible for the processing of inflammatory response, in part by the release of ROS [58]. Colchicine treatment leads to remission in FMF patients due to its effect of reducing/protecting oxidative stress, stabilizing the antioxidant redox system, and releasing Ca2+ from neutrophils into the serum [59]. However, there is also evidence that colchicine itself causes oxidative stress [60].

This study has important limitations that should be mentioned when interpreting the results. First, the non-randomized design with no control group precludes a firm conclusion being drawn from the study. However, all observations were paired to the individual patient baseline observations, so each patient served as their own control. Moreover, the observed responses to the bioactive nutrient combination are highly significant and not observed in the natural history of these diseases (where patients rarely improve spontaneously), that it is difficult to adjudicate these observations to chance alone. While a placebo-controlled study would be optimal to confirm our observations, we believe that our findings support a clinically important effect of the AAL, AIC, and AOL on inflammation, endothelial function, and oxidative state. In particular, the reduction in proteinuria is striking and has the potential to greatly affect patient outcomes long term. Second, although the number of patients enrolled was small, this is one of the largest cohorts of FMF patients with AA amyloidosis in the world. The power to detect relatively small effects is limited with small cohorts such as ours, but we believe that our observations are of importance when randomized controlled trials are designed. Studies with significantly larger cohorts are unlikely to be feasible in a rare disease such as FMF. 

## 5. Conclusions

In conclusion, treatment with a combination of bioactive nutrients for 24 weeks was significantly associated with reductions in laboratory biomarkers of systemic inflammation, enhancement of endothelial functions, and oxidative status. Our results show the association among these partners with the development of atherosclerosis and kidney injury in patients with AA renal amyloidosis secondary to FMF. Effective management of the inflammatory process, endothelial cell dysfunction, and oxidative markers may provide long-term cardiovascular and renal benefits in this rare patient group. 

## Figures and Tables

**Table 1:** Characteristics features of included patients for the study.

Demographic and Clinical Features	
Gender (male, n, %)	52 (77.6%)
Current age, year (median, range)	36 (21–66)
Age at FMF diagnosis, year (median, range)	16 (6–26)
Age at Amyloidosis diagnosis, year (median, range)	24 (17–48)
BMI (kg/m^2^) (mean, SD)	25.3 ± 2.4
Systolic blood pressure (SBP) (mmHg) (mean, SD)	139 ± 15
Diastolic blood pressure (DBP) (mmHg) (mean, SD)	86 ± 9
Colchicine usage (n, %)	56 (83.6%)
**MEFV Alleles frequency (n, %)**	
M694V	97 (74%)
M680I	13 (9.9%)
E148Q	11 (8.4%)
V726A	5 (3.8%)
M694I	4 (3.1%)
L695A	1 (0.8%)
Total	131 (100%)

**Table 2:** Baseline clinical features.

Clinical Feature (n = 67)	n (%)
Fever	59 (88.1)
Abdominal pain	46 (68.7)
Arthritis	45 (67.2)
Chest pain	33 (50.3)
Arthralgia	26 (38.8)
Myalgia	17 (25.3)
Erysipelas like erythema	16 (23.9)
Diarrhea	11 (16.4)
Vomiting	8 (11.9)
Protracted febrile myalgia	5 (7.5)
Pericarditis	3 (4.5)
**Complications (n = 67)**	
Appendectomy	29 (43.3)
Splenomegaly	16 (23.9)
Peritoneal adhesion	5 (7.5)
Intestinal occlusion	5 (7.5)
Thrombosis	3 (4.5)
Acute orchitis	3 (4.5)

**Table 3:** Baseline clinical and laboratory characteristics of patients and longitudinal changes following 24 weeks of AAL, AIC, and AOL therapies.

	AAL, AIC and AOL Therapies (n = 67)			
	Baseline	24th Week	∆	*p*
FMD (%)	5.0 ± 0.7	6.4 ± 0.8 **	1.3 ± 0.9	<0.001
CIMT (mm)	0.9 ± 0.2	0.7 ± 0.1	−0.2 ± 0.2	<0.001
hs-CRP (mg/L)	25.5 (4.4–48.0)	3.0 (1.0–9.1) *	−21.6 (−11.4–0.4)	<0.001
PTX3 (ng/mL)	13.4 (2.3–67.0)	2.3 (0.4–14.5) *	−10.4 (−66.2–3.14)	<0.001
Serum Amyloid A (mg/dL)	6.6 ± 2.2	2.7 ± 1.7	−3.8 ± 2.6	<0.001
Total Cholesterol (mg/dL)	221.2 ± 60.3	155.8 ± 35.4 **	−65.3 ± 55.5	<0.001
Triglycerides (mg/dL)	145.7 ± 36.7	139.7 ± 20.3 **	−6.1 ± 34.5	0.15
LDL-cholesterol (mg/dL)	131.7 ± 26.4	119.7 ± 17.8 **	−12.1 ± 23.5	<0.001
HDL-cholesterol (mg/dL)	38.8 ± 6.2	45.4 ± 4.6 **	6.6 ± 7.6	<0.001
eGFR (mL/min/1.73 m^2^)	110.2 ± 12.8	104.1 ± 11.2 **	−6.1 ± 11.9	<0.001
HOMA-IR	1.6 ± 0.8	1.2 ± 0.4 **	−0.44 ± 0.87	<0.001
Serum albumin (g/dL)	3.6 ± 0.2	4.0 ± 0.4 **	0.4 ± 0.5	<0.001
MDA (nmol/mL)	4.2 ± 1.8	1.8 ± 0.5 **	−2.2 ± 1.8	<0.001
CuZn-SOD (U/mL)	431.5 ± 154.7	538.1 ± 146.4 **	159.7 ± 211.8	<0.001
GSH-Px (U/mL)	47.8 ± 13.2	74.1 ± 20.3 **	26.3 ± 21.1	<0.001
ADMA (µmol/L)	4.5 ± 2.6	1.3 ± 0.6 **	−3.2 ± 2.5	<0.001
Proteinuria (mg/24 h)	6856 ± 3117	4090 ± 2360	−2776 ± 2875	<0.001

eGFR, estimated glomerular filtration rate; HOMA, homeostasis model assessment; MDA, Malondialdehyde; CuZn-SOD, copper zinc-superoxide dismutase; GSH-Px, glutathione peroxidase; hsCRP, high sensitivity C reactive protein; PTX3, pentraxin 3; ADMA: asymmetric dimethyl arginine; FMD, endothelium dependent vasodilatation; CIMT, carotid intima media thickness. *Morinda citrifolia* (anti-atherosclerotic liquid—AAL) (3 mL once per day); omega-3 (anti-inflammatory capsules—AIC) (3 capsules once per day) extract with Alaskan blueberry and 21 different red purple fruit vegetables (anti-oxidant liquid—AOL) (30 mL once per day); ** paired samples *t*-test, * Wilcoxon test; data are means ± SD and median (min–max).

**Table 4:** Correlations between changes of FMD, proteinuria, CIMT, cholesterol, hsCRP, PTX3, and ADMA.

Pearson Correlation		∆FMD	∆Proteinuria	∆CIMT	∆Cholesterol	∆MDA	∆hsCRP	∆ptx3
∆proteinuria	r	−0.533	1					
	*p*	<0.001						
∆CIMT	r	−0.373	0.320	1				
	*p*	0.002	0.008					
∆Cholesterol	r	−0.263	0.479	0.389	1			
	*p*	0.031	<0.001	0.001				
∆MDA	r	−0.384	0.409	0.485	0.407	1		
	*p*	0.001	0.001	<0.001	0.001			
∆hsCRP	r	−0.476	0.414	0.460	0.554	0.648	1	
	*p*	<0.001	<0.001	<0.001	<0.001	<0.001		
∆PTX3	r	−0.497	0.363	0.477	0.563	0.682	0.652	1
	*p*	<0.001	0.003	<0.001	<0.001	<0.001	<0.001	
∆ADMA	r	−0.485	0.406	0.440	0.577	0.693	0.562	0.717
	*p*	<0.001	0.001	<0.001	<0.001	<0.001	<0.001	<0.001

FMD, endothelium dependent vasodilatation; CIMT, carotid intima media thickness; MDA, malondialdehyde; hsCRP, high sensitivity C reactive protein; PTX3, pentraxin 3; ADMA: asymmetric dimethyl arginine.

## Data Availability

All data relevant to the study are included in the article. Data described in the manuscript, code book, and analytic code will be made available upon request.
